# Why don’t we have an effective tuberculosis vaccine yet?

**DOI:** 10.1586/14760584.2016.1170599

**Published:** 2016-05-03

**Authors:** Tamara Davenne, Helen McShane

**Affiliations:** ^a^ The Weatherall Institute of Molecular Medicine, University of Oxford, John Radcliffe Hospital, Oxford, UK; ^b^ Jenner Institute, University of Oxford, Oxford, UK

**Keywords:** *Mycobacterium tuberculosis*, vaccine, BCG, variability, model, protection

## Abstract

*Mycobacterium tuberculosis* (*M.tb*) has co-evolved with humans for thousands of years, to cause tuberculosis (TB). The success of *M.tb* as a pathogen is in part because of the ways in which *M.tb* evades and exploits different cell subsets, to persist and cause disease. *M.tb* expresses numerous molecules to prevent its recognition and destruction by immune cells. The only licensed vaccine against TB, Bacillle Calmette-Guerin (BCG), is effective at preventing disseminated disease in infants but confers highly variable efficacy against pulmonary TB in adults, particularly in the developing world. A greater understanding of the reasons for this variability, together with a better understanding of the early, innate, and non-antigen specific mechanisms of protection would facilitate the design and development of more effective vaccines.

Tuberculosis (TB) killed 1.5 million people worldwide in 2014 despite the availability of bacillus Calmette–Guérin (BCG), the only current licensed vaccine against TB, first developed almost a century ago [1]. The human-*Mycobacterium tuberculosis (* *M.tb)* host–pathogen interaction has evolved for thousands of years and *M.tb* expresses a plethora of antigens to counteract its recognition, phagocytosis, and destruction by immune cells. The efforts made in the past decades to better understand this interaction have allowed the identification of key mycobacterial proteins and the development of more than 15 vaccines currently being evaluated in clinical trials [1]. Although our understanding of TB pathogenesis is improving, many questions remain unanswered. This review will focus on the limitations of the current BCG vaccine, the mechanisms used by *M.tb* to survive within alveolar macrophages (AMs) and how these mechanisms may be disrupted through novel vaccination strategies. The concept of trained innate immunity will be discussed, together with the potential implications of this for the development of an effective TB vaccine.

## Limitations of BCG vaccine

TB remains the first cause of death from a single infectious agent despite the availability of the BCG vaccine [1]. TB still causes more than a million deaths per year in spite of a 47% drop in TB mortality rate since 1990 [1]. Although BCG is protective against disseminated disease in young children, it has variable efficacy against pulmonary TB, particularly in adults [2–5]. A more consistently effective vaccine than BCG in both adolescents and adults is needed to achieve the ‘End TB strategy’ set by the World Health Organization. BCG is an attenuated strain of *Mycobacterium bovis*. The loss of virulence of this strain is caused by the deletion of the RD-1 locus that encodes nine genes including a 10-kDa cultured filtered protein (CFP-10) and a 6-kDa early secreted-antigen (ESTAT-6) [6]. Both are proteins secreted by the Snm secretion system and are considered essential virulence factors contributing to *M.tb* pathogenesis, suggesting that they may be good vaccine targets [7]. ESAT-6 was shown to block TLR2 at the surface of the macrophage and CFP-10/ESAT-6 complex was shown to downregulate reactive oxygen species (ROS) production [8,9]. In addition, CFP-10/ESAT-6 complex was shown to dissociate under acidic condition allowing ESTAT-6 to destabilize and lyse liposomes [10]. The lack of expression of CFP-10 and ESAT-6 by *Mycobacterium bovis* in the BCG vaccine prevents the bacteria to counteract its destruction by the host cell, allowing it to kill *M.tb* efficiently. The destruction of the pathogen makes *M.tb* antigens available allowing the subsequent activation of CD4 and CD8 cells via antigen presenting cells and the production of Interferon-γ (IFN-γ), a key cytokine in the immune response against *M.tb* [11].

There are several factors that could explain the variable efficacy of BCG. Several decades ago, the strain of BCG used for vaccination was sent to different laboratories worldwide for vaccine production. Over time, hundreds of passages and differences in BCG growth protocols between laboratories have contributed to genetic variability among the strains [12]. While it is clear that there are genetic differences between the different BCG strains currently in clinical use, it is less clear how this impacts on efficacy. A mouse study suggests that different BCG strains have considerable differences in immunogenicity and that these differences may play a role in BCG efficacy [13]. However, a recent meta-analysis showed no relationship between the estimated vaccine efficacy and the different BCG strains used in clinical trials over different years [14]. In addition, it has been observed that the culture media used to grow BCG could impact vaccine immunogenicity, and that BCG cultured in Sauton media was more persistent in macrophages, more effective at inhibiting apoptosis and induced stronger inflammatory responses than when cultured in Middlebrook 7H9 medium [15]. Although it is possible to demonstrate differences in immunogenicity, without an immune correlate of protection, it is difficult to extrapolate these to meaningful differences in efficacy. Another potential explanation for the variable efficacy conferred by BCG against pulmonary disease is that exposure to non-tuberculous mycobacteria interferes with BCG efficacy, either by masking or by blocking. The masking hypothesis is demonstrated by studies where BCG-naïve adolescents in London and Malawi were vaccinated with BCG. The children in London had low baseline mycobacterial immunity which was significantly increased after BCG vaccination. In contrast, the children in Malawi had high background responses and little incremental increase after vaccination [16]. This data suggests that prior immunity induced by non-tuberculous mycobacteria masks the effects of BCG. The blocking hypothesis suggests that background immunity induced by non-tuberculous mycobacteria might inhibit the replication of BCG, which is necessary for efficacy, and therefore the ‘take’ of BCG [17].

The limitations and causes of BCG variability are still not fully understood and our struggle to improve BCG is in part due to our lack of understanding of what determines the outcome of *M.tb* infection. Attempts to develop better vaccines continue. It is important to retain the protective efficacy conferred by BCG against disseminated disease, and strategies to develop a better vaccine include developing improved strains of BCG, or alternative whole mycobacterial priming vaccines based on attenuated strains of *M.tb*, and developing subunit booster vaccines, to be administered after a BCG priming immunization [18]. The result of the recent phase IIB clinical trial of the vaccine candidate MVA85A, designed to boost BCG efficacy in infants, was disappointing [19]. The MVA85A vaccine was well tolerated and modestly immunogenic but did not confer significant protection against TB disease or *M.tb* infection in this age group. The lack of a validated immune correlate of protection, together with uncertainty as to which animal model, if any, best represents human disease, means vaccine development and predicting, which candidate vaccine might protect in humans is very challenging. The animal models are necessarily simplified models and age group, gender, ethnicity, previous exposure to mycobacteria, other co-infections including HIV, and helminths, may all impact on immunogenicity and protective efficacy. These variables are very difficult to mimic in animal models. Trials with MVA85A show high levels of immunogenicity in UK adults but poor levels of immunogenicity in South African infants, demonstrating the variability of vaccine response in different populations. Reasons for this variability need to be better understood. An animal model that reflects better the diversity of human populations would be ideal in order to focus valuable resources in future clinical trials on vaccines most likely to be protective in humans.

A different type of vaccination should also be considered in order to better mimic the natural route of *M.tb* infection in the lungs and thus to induce a better immune response, a short review was recently published on this topic [20]. The first aerosol vaccine clinical trial against *M.tb* was reported in 2014, and there are more studies underway. Ultralow dose *M.tb* challenge were performed in non-human primates and showed different outcomes between rhesus and cynomolgus macaques [21], underlying the importance of the choice of the animal model. Aerosol vaccine alone or in combination with other routes of immunization may improve immunogenicity against *M.tb* by directly targeting and training AMs to subsequent infections.

An effective TB vaccination strategy remains an important need for public health. We must understand the limitations of the current BCG vaccine and consider the variables that influence the outcome of vaccination and how they impact on future vaccine design. In addition, it is important to be able to design booster vaccines, which are highly potent and capable of inducing a strong immune response that overcomes differences in genetic background, ethnicity, and prior mycobacterial exposure between individuals [19]. Identifying *M.tb* virulence factors may lead to the identification of new vaccine targets able to induce strong T and B cell responses. Our knowledge of the host–pathogen interaction has increased significantly during the past decades, but it is still not completely clear what defines an efficient immune response against *M.tb*.

## Mycobacterium tuberculosis counteracts the innate immune response

*M.tb* is transmitted by airborne droplets from individuals with smear-positive pulmonary disease by coughing, sneezing, singing, or talking [22]. *M.tb* reaches the lung alveoli where it is taken up by resident AMs, dendritic cells (DCs) and other phagocytic cells. AMs are unique mucosal immunoregulatory cells that express various pattern recognition receptors (PRRs), and are the preferred *M.tb* target cell for uptake [23]. The mycobacterial pathogen associated molecular patterns (PAMPs) are recognized by PRRs expressed at the surface of the AMs. The PRRs involved in *M.tb* detection are the Toll-like receptors, Fcγ receptors, complement receptors, and PRRs, such as C-type lectin mannose receptors, dectin-1 and scavenger receptors [24]. Once the inhaled *M.tb* has been engulfed by AMs, there is a spectrum of clinical outcome which includes (i) clearance: the pathogen will be cleared by the immune system, (ii) primary TB disease: the bacteria grow and multiply after infection, ultimately causing disease, (iii) latent *M.tb* infection: the bacilli become dormant and may never cause the disease, and (iv) reactivation: the latent bacilli reactivate at a point in time distant to the primary infection [25,26]. Clearance of the pathogen is estimated to occur in up to 90% of cases although the immunological mechanisms responsible are not clearly defined [27].

*M.tb* has evolved different mechanisms to evade recognition by immune cells. A recent study demonstrated that *M.tb* expresses cell surface-associated phthiocerol dimycoceroserate (PDIM) lipids to mask the underlying PAMPs [28]. The same group also showed that related phenolic glycolipids expressed by *M.tb* promote the recruitment of macrophages through a host chemokine receptor 2 (CCR2) pathway [28]. The eventual fate of *M.tb* is to be phagocytosed following its binding to receptors at the surface of the macrophages. Pathogens phagocytosed by a macrophage will typically be processed by the endocytic pathway, a process which involves the fusion of the phagosome with lysosomes. The bacterium is trapped within the acidic phago-lysosome which subsequently leads to its elimination by enzymes, a process called lysosomal degradation. Here again, *M.tb* has developed different strategies to circumvent this process, thus facilitating persistence within the host cell. A recent study performed using guinea pig tissue confirmed that *M.tb* acid phosphatase (SapM) dephosphorylates phosphatidylinositol 3-phosphate (PI3P) present on the phagosome leading to the arrest of phagosomal maturation [29]. Another protein was shown to modify the host signaling pathway; the tyrosine phosphatase PtpA dephosphorylates and inactivates the host vacuolar protein sorting-VPS33B preventing phagosome–lysosome membrane fusion [30]. More recently, PtpA binding to ubiquitin was shown to dephosphorylate phosphorylated Jnk and MAPK 38, thus suppressing inflammatory responses through the MAPK–JNK pathway [31]. Moreover, the host endosomal sorting complexes required transport (ESCRT) machinery involved in phagosome maturation is disrupted by EsxH, an *M.tb* virulence factor secreted by the Esx-3 type VII secretion system (TSSS) [32]. The *M.tb* cell envelope is rich in lipids and carbohydrates, including lipoglycans such as lipoarabinomannan (LAM). LAM has been shown to prevent phagosome-lysosome fusion and the distribution of LAM within the cell envelope is controlled by the lipoprotein LprG [33,34]. This supports the idea that *M.tb* cell wall lipids act as virulence factors during infection; mycobacterial virulence lipids have been reviewed in detail in Ref. [35].

More recently another study showed that *M.tb* nucleoside diphosphate kinase (Ndk) contributes to *M.tb* virulence via attenuation of NADPH oxidase-mediated host innate immunity. Ndk inactivates GTPase Rac1 leading to the inhibition of NADPH oxidase 2 (NOX2) assembly, which is required for apoptosis and the production of ROS, an antibacterial effector also involved in signaling pathways [36,37]. *M.tb* has also been shown to counteract the ROS produced by the host as a defense mechanism against bacteria and fungi [38]. This is supported by another study that shows the interaction between *M.tb* and the macrophage mitogen-activated protein kinase (MAPK) via RecA to control ROS and reactive nitrogen species (RNS) production in infected AMs [39].

*M.tb* also uses soluble secondary messengers (intracellular signaling molecules that trigger various mechanisms) to manipulate the host. Cyclic adenosine monophosphate (cAMP) is involved in bacterial signaling pathways, where it binds to the transcription factor cAMP-receptor protein (Crp) and regulates the transcription of genes involved in glucose response, biofilm regulation, type III secretion system, quorum sensing and virulence-gene expression [40]. A commonly used laboratory strain of *Mtb*, H37Rv, expresses 16 adenylyl cyclases (ACs) that increase the level of cAMP within the host leading to the disruption of phagosome assembly and maturation within macrophages [41,42]. A recent study suggests that high cAMP levels prevent autophagy [43], an important mechanism involved in the destruction of intracellular pathogens, through cargo sequestration to phagosomes that fuse with lysosomes [44,45].

The interaction of *M.tb* with the host cell leads to an innate and adaptive immune response against *M.tb* which leads to the subsequent formation of a granuloma, a complex structure formed of an accumulation of inflammatory immune cells aiming to contain the pathogen. Granuloma formation is caused by the recruitment of uninfected local macrophages, neutrophils, monocytes from the blood and the later T-cell infiltration [46]. The process by which granuloma are formed following *M.tb* infection was described in a recent review [47]. Although the macrophage is the first cell line of defense, T-cell mediated immunity plays a major role in the subsequent defense against *M.tb* by the secretion of IFN-γ, one of the main mediators of macrophage activation [48,49].

## Can innate immunity be trained to target ***M.tb***?

Despite conferring variable efficacy against TB, BCG has been observed to have a non-specific protective effect against other pathogens. The early studies with BCG show that the protective effect of the vaccine exceeded the disease burden in the related age group [50–52]. It was shown that the non-specific effect of BCG was observed even in T- and B-cell deficient mice infected with *Schistosoma mansoni* [53]. Macrophages pre-exposed to BCG (‘trained macrophages’) displayed an increased PMA-induced production of H_2_O_2_ and enhanced phagocytosis [54]. A recent study performed with human cells demonstrated that macrophages undergo long-term epigenetic programming upon β-glucan and lipopolysaccharide-induced training [55]. Epigenetic reprogramming and cross-reactivity of the immune response may explain the beneficial health-related effect of BCG vaccination.

Innate immunity has always been considered as non-antigen-specific immunity involving different cell types and specific germline-encoded receptors able to recognize various common PAMPs. Innate immunity does not generate long-term protective immunological memory in contrast to the adaptive immune response [56]. In recent times, this paradigm has begun to shift as emerging data demonstrates that macrophages and NK cells can be ‘trained’ through epigenetic reprogramming and become more efficient upon secondary infection. Macrophages are the main target cells of *M.tb* and the bacteria have evolved various virulence strategies to evade recognition. Efficiently training macrophages prior to *M.tb* infection may be the key to allow them to clear the bacteria. Most vaccine immunogenicity studies are focused on evaluating the adaptive immune response, and perhaps the potential of a vaccine to train macrophages should also be assessed. A better understanding of this phenomenon may help to develop better vaccines against TB and could potentially help to develop vaccines against other intracellular pathogens.

## Expert commentary

A better vaccine against tuberculosis is urgently needed. Research over the past decades has identified *M.tb* virulence factors and its interactions with macrophages, helping to identify potential vaccine targets. There are currently several new vaccines or boosts in clinical trials, these are expressing different *M.tb* antigens in various vectors and are administered through different routes. This reflects the progress recently made in vaccine design – thanks to a better understanding of the interaction of *M.tb* with the players of the immune system at a molecular level. Possible explanations for the variable efficacy of BCG include BCG strain and previous exposure to environmental mycobacteria. These parameters need to be considered in future vaccine design, and experiments in animal models should be designed to best reflect efficacy within a specific human population. Indeed, the lack of correlation of protection in human and animal models makes it very difficult to efficiently evaluate whether an immune response against new vaccines will be protective against *M.tb* or not. The possibility to train macrophages to increase efficiency of eliminating *M.tb* should be explored. Using aerosol vaccines could be the key for a better immunization, this will reproduce the natural route of infection of *M.tb*, targeting directly AMs. More information is required on how durable innate immune memory is, and to determine how it could be exploited in future vaccine design.

## Five-year view

Progress has been made in the field of TB research in the past decades but there are still significant challenges that need to be overcome in order to expedite the development of more potent vaccines against TB in the next few years. Molecular mechanisms by which *M.tb* blocks its destruction by AMs can be dissected and this will give us more information about what defines the outcome of the infection. In addition, the development of animal models that predict more accurately the heterogeneity of the immune response against *M.tb* in human, and the identification of new markers that correlates with protection should allow better prediction of vaccine efficacy. There are currently 16 vaccine candidates being evaluated in clinical trials, reflecting the progress that has been made in this field. A greater understanding of the underlying immunology of *M.tb* infection will contribute to the design and development of an effective TB vaccine.

## Key issues

- Tuberculosis is the leading cause of death by an infectious pathogen, killing more than one million of people per year.
- It is currently estimated than one third of the population is latently infected with *M.tb*.
- The only licensed TB vaccine, Bacillus Calmette-Guerin, has shown variable efficacy.
- *Mycobacterium tuberculosis* expresses a multitude of proteins which help counteract its killing by alveolar macrophages.
- The underlying reasons for the variable efficacy of BCG are not clear, making the design of a new replacement or booster vaccine challenging.
- The lack of animal models that adequately reflect the human heterogeneous response against *M.tb* makes preclinical prediction of human vaccine efficacy difficult.
- A better understanding of the variable efficacy of BCG and the complex host–pathogen interaction is key to the development of a better vaccine.
- BCG has been demonstrated to confer some non-specific protective effects against other pathogens. This may be due to the potential of macrophages to be ‘trained’ by BCG.
